# Survey of public knowledge, attitudes, and practices regarding personal protection against COVID-19 in the post-pandemic era

**DOI:** 10.3389/fpsyg.2024.1411055

**Published:** 2024-06-10

**Authors:** Shixian Luo, Jing Xie, Jie Chen, Hongyu Li, Sining Zhang

**Affiliations:** ^1^School of Architecture, Southwest Jiaotong University, Chengdu, China; ^2^Graduate School of Horticulture, Chiba University, Chiba, Japan

**Keywords:** COVID-19, KAP survey, post-pandemic era, risk management, vulnerable populations, non-pharmaceutical interventions

## Abstract

In the emerging post-pandemic era (the ‘wavelet’ era), humans must coexist with viruses for the foreseeable future, and personal protective behaviors will largely replace national-level preventive measures. In this new normal, encouraging the public to implement proper personal protective behaviors against the coronavirus disease (COVID-19) is vital to the sustainable development of cities and communities. This knowledge–attitude–practice (KAP) survey conducted in Chengdu (*N* = 900) narrowed the knowledge gap regarding post-pandemic public practices of protective behavior. Findings show that:(1) approximately 1/3 of the respondents are currently not concerned about COVID-19 at all; (2) respondents with different demographics and individual COVID-19-related factors showed significant differences in practice behaviors indoors and outdoors; (3) vulnerable groups performed better in practice behavior indoors/outdoors; (4) because the public may relax their vigilance outdoors, public places may become a transmission threat in the next outbreak; (5) attitudes are important, but limited incentives for practice; and (6) when knowledge increases beyond a threshold (68.75–75% in this study), protective behaviors decrease. Our results suggest that authorities must continue to educate and motivate the public, extending measures to cover personal protective practices, and have targeted policies for specific demographics to ensure equity in healthcare in the event of another pandemic (COVID-19 and alike crisis). Besides, comparing the results of the current study with similar studies conducted in other parts of the world can provide insights into how different populations respond to and adopt COVID-19 protective behaviors. The epidemiologists can use the data collected by this and other KAP surveys to refine epidemiologic models, which can help predict the spread of the virus and the impact of interventions in different settings.

## 1 Introduction

**FIGURE 1:**
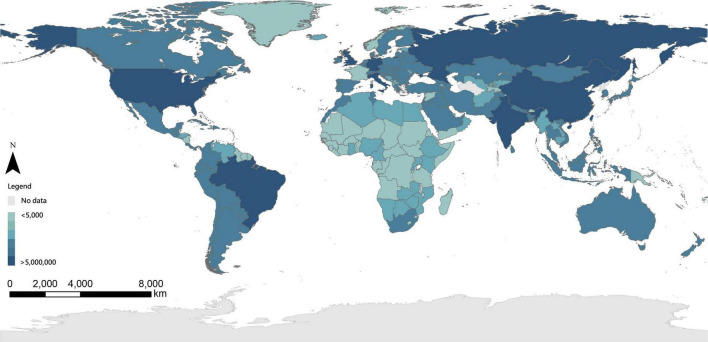
Global reported cases as of May 10, 2023 (data resource: World Health Organization, 2023).

On 31 December 2019, a case of “viral pneumonia” was first reported in Wuhan, Hubei Province of China (National Health Commission of the People’s Republic of China, 2020). Thereafter, the coronavirus disease 2019 (COVID-19) raged across the world for three years until 5 May 2023, when the World Health Organization (WHO) declared the end of the emergency phase of COVID-19 (Lenharo, 2023). The COVID-19 pandemic has revolutionized the daily lives of people worldwide (like the interruptions to work schedules, insecure food supplies, Diotaiuti et al., 2023), resulting in record numbers of cases and deaths (Wang et al., 2022; Chow et al., 2023), and creating a huge healthcare burden globally (Figure 1).

During the pandemic, non-pharmaceutical interventions (NPI) were introduced by governments as the first public health response to impede the spread of infection and reduce the healthcare burden (Chow et al., 2023; Santos et al., 2023), for example, in Sri Lanka (Jayaweera et al., 2021), Sweden (Sjödin et al., 2020), China (Lai et al., 2020), UK (Davies et al., 2020), and Saudi Arabia (Bisanzio et al., 2022). According to the European Centre for Disease Prevention and Control (2023), NPI involves three main levels: individual (hand washing, wearing a mask), environmental (ventilation of indoor spaces), and population (physical distancing and movement restriction). Some countries have incorporated additional levels such as ‘community’ (e.g., shutting facilities, canceling gatherings, stay-at-home; Chow et al., 2023). These measures aimed to control the spread of the virus by delaying the peak of the outbreak and reducing its magnitude, thus allowing time for preparing the healthcare system (Lai et al., 2020). However, despite the rollout of the COVID-19 vaccine and its initial success, its efficacy is being challenged by the evolution of SARS-CoV-2 and emergence of viral variants (Jacobs et al., 2023). Moreover, some potential drugs for the treatment of COVID-19, such as Lopinavir-ritonavir, and Nafamostat, have been reported to have side effects (Cao et al., 2020; Sonawane et al., 2020). Consequently, many countries continue to utilize NPI as effective measures against COVID-19 (Santos et al., 2023).

**FIGURE 2:**
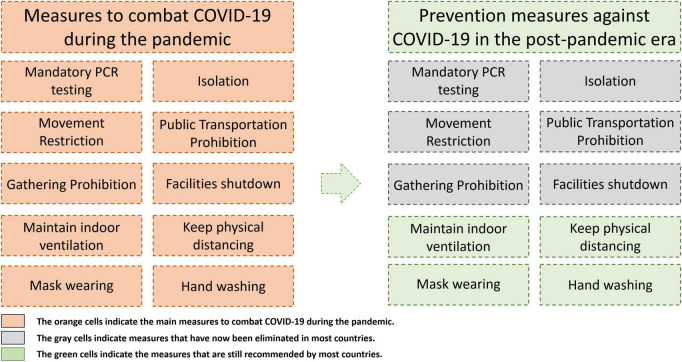
Preventive measures against COVID-19 during the pandemic and in the post-pandemic era.

The COVID-19 pandemic has been effectively contained, with a significant decrease in the number of cases and deaths as a result of global efforts (Lenharo, 2023). Today, three years after the start of the pandemic, SARS-CoV-2 shows no signs of a seasonal pattern similar to that of influenza (Callaway, 2023). However, infections are still on the rise in some countries due to new variants (e.g., XBB.1.16) and diminished vaccine efficacy, leading to a new normal in the post-epidemic era—the ‘wavelet’ era (Callaway, 2023). This phase is characterized by frequent, less deadly waves and a higher proportion of mild infections (Callaway, 2023). The world will remain in the midst of the pandemic for a prolonged period, and this new status does not mean that countries can be incautious. There is an urgent need for countries to bolster their preparedness for future pandemics (Lenharo, 2023). In addition, as of 5 May 2023, many countries have eliminated strict polymerase chain reaction (PCR) testing, quarantine, and movement restrictions. In some countries, citizens can decide whether to implement government-recommended NPI measures such as wearing masks (Joshi and Wende, 2023; The Japan Times, 2023). Therefore, it is important to acknowledge that personal protective behaviors in the post-pandemic era will replace national-level precautionary measures (Figure 2). Moreover, it is essential for individuals to adopt NPI (e.g., washing hands, wearing a mask, maintaining physical distance) to reduce their own risk of infection, because these guidelines are easy, inexpensive, and effective.

Although wavelets do not always cause spikes in hospitalizations and deaths, their health impact varies across countries (Callaway, 2023), suggesting that the world could still encounter another COVID-19 outbreak, although the probability is currently low. Moreover, it is difficult to completely eliminate this threat in a short time, and humans must coexist with COVID-19 (Rudan et al., 2022; Song et al., 2022; Su et al., 2022). Studies on previous outbreaks suggest that public awareness plays an important role in curtailing the infection spread (Lau et al., 2003; Yap et al., 2010), and inadequate knowledge often leads to careless attitudes and inappropriate practices, exacerbating risk (Sujarwoto et al., 2022). Therefore, in the post-pandemic era, it is important for managers and policymakers to promote knowledge of personal prevention and encourage attitudes and practices that reduce the risk of COVID-19 spreading.

To date, several knowledge–attitudes–practices (KAP) surveys on COVID-19 have been conducted, such as Zhong et al. (2020), Feldman et al. (2021), Chai et al. (2022), Sujarwoto et al. (2022), Qalati et al. (2023). KAP surveys aim to determine what the public knows (knowledge), believes (attitudes), and does (practices) with regard to a particular topic of interest. Attitudes reveal the survey respondents’ propensity to act on the topic, and practices refer to the measures/behaviors adopted in response to it (Chai et al., 2022). Thus, KAP intervention is considered a key public health strategy for controlling diseases by changing citizens’ health behavior (Sujarwoto et al., 2022). However, KAP investigations were conducted during the COVID-19 pandemic, and none have examined the post-pandemic era. Furthermore, these studies mainly dealt with general knowledge regarding COVID-19, such as the incubation period (Qalati et al., 2023), clinical symptoms (Feldman et al., 2021), route of infection (Zhong et al., 2020), and isolation measures (Chai et al., 2022). Although the value of this information is undeniable, its usefulness is limited in the post-pandemic era context, as the public needs to know more about proper personal protection; also, the KAP survey can be used to assess the public’s strategies to protect themselves from specific disasters (Rahman et al., 2023).

Therefore, this KAP survey on personal protection in the post-pandemic era is relevant in reducing infection risk and developing public health policies. Moreover, the items in this survey were informed by the most recent literature and policy documents, rendering it useful as a reference for future studies. Last but not least, in line with United Nations Sustainable Development Goal 11, by 2030, to significantly reduce the number of deaths and the number of people affected, to significantly reduce the negative impacts of disasters and to focus on the protection of the poor and the vulnerable; and, furthermore, to make cities and human settlements inclusive, safe, resilient and sustainable. Therefore, another potential value of this KAP survey is to provide recommendations to city managers on pandemic control related education/training to meet the needs of resilient cities for a healthy population. In summary, the specific objectives of this study are to:

(1) To investigate whether public KAP behaviors regarding COVID-19 personal protection are adequate in the post-pandemic era;
(2) To discuss the sociodemographic characteristics that influence the three dimensions of KAP;
(3) To discuss the influence of individual status on the three dimensions of KAP, such as whether one has been infected with COVID-19, current concerns about the pandemic, and perceived risk of infection;
(4) To analyze how the three KAP dimensions relate to each other.

## 2 Methodology

### 2.1 Study area

**FIGURE 3:**
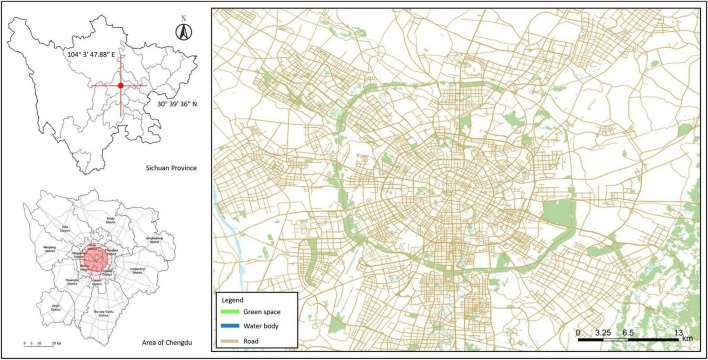
The study area.

A cross-sectional survey was conducted in Chengdu (alternatively romanized as Chengtu), the capital of Sichuan Province, located on the western edge of the Sichuan Basin, with an area of 14,335 km2 and a population of over 20 million (Figure 3; Chengdu Statistical Yearbook, 2021). It is the political, industrial, cultural, logistical, and scientific center of the province, and an important economic center for the entire southwest region (Qin, 2015). Chengdu is a megacity and the core of the Chengdu–Chongqing Urban Agglomeration, identified by the Chinese State Council as an important central city in western China (Yang L. et al., 2022). Another important reason for choosing Chengdu as the study area is that it is one of the most affected by the COVID-19 pandemic and representative of cities in southwest China (Shi et al., 2023). On 7 December 2020, several areas in the city were upgraded to intermediate risk due to local transmission. In order to control the epidemic, the Chengdu city government quickly took decisive and strict action to effectively block further spread of COVID-19, clearing all the intermediate risk areas by December 31 (Jiang et al., 2022). Communities in Chengdu were strictly managed according to the WHO and China’s prevention and control management measures until April 2021 to prevent the spread of the infection (Yang K. et al., 2022). From December 2022, the National Health Commission of the People’s Republic of China announced the rollout of new prevention and control standards: no health code checks, no quarantine, and no strict management measures, such as movement restrictions, within the country, and residents’ lives began to return to normal (National Health Commission of the People’s Republic of China, 2023). Thus, Chengdu has entered the post-pandemic era (Jin et al., 2023).

### 2.2 Study population and data collection

Questionnaire responses were collected from Chengdu residents between 20th to 30th April 2023, using the Credamo online questionnaire platform.^1^ Credamo is a professional online questionnaire service with over 3 million Chinese adults registered across all provinces in China (all users have been authenticated by Credamo) and has proven to be a reliable for collecting valid data in several prior studies (He et al., 2023; Xiao et al., 2023). The Credamo platform was used to send job advertisements to participants who met the study requirements (residing in Chengdu, 18 years of age or older), and those interested completed an online survey. Informed consent was obtained before the participants answered the online questionnaire. To avoid invalid and duplicate responses, two screening procedures were implemented: (1) platform-provided “CAPTCHA (Completely Automated Public Turing test to tell Computers and Humans Apart)” questions were used, which are easy for humans but extremely difficult for bots or scripts. Based on the “CAPTCHA” results, the online survey of participants who did not pass was automatically terminated by the platform; (2) the same IP address could not access the survey more than once. Ultimately, 993 responses were submitted; however, 93 were removed due to abnormal response times (< 120 s or > 600 s), leaving a sample of 900 (90.6%) for subsequent analysis. The minimum sample size required was calculated using the formula in Fecso et al. (1996).


(1) $$n=\frac{z^{2}\,\left(p\right)\,\left(1-p\right)}{c^{2}}$$

Where *n* = sample size;

*z* = Z-value (e.g., 1.96 for 95% confidence level);

*p* = percentage of choosing an option; when *p* = 0.5, the sample size *n* is maximized;

*c* = confidence interval (also error tolerance), expressed as decimal (e.g., 0.05 = ±5).

According to Equation (1), the minimum sample size required is 385 when the confidence level is 95.0% (*z* = 1.96), *p* = 0.5, and *c* = 5% (Fecso et al., 1996). Therefore, the sample size of 900 used in this study was adequate. The study was approved by the academic committee of the first author’s institution, and the ethical review was waived because the study was non-invasive and all participants remained anonymous.

### 2.3 Survey instruments

The online questionnaire comprised three sections. The first consisted of respondents’ sociodemographic characteristics (gender, age, marital status, type of residential area, education level, occupation, and monthly income), which have been previously shown to influence all three dimensions of KAP (Chai et al., 2022; Sujarwoto et al., 2022; Qalati et al., 2023; Rahman et al., 2023). The second section enquired about individual status in the post-pandemic era, such as whether one had been previously infected with COVID-19, current concerns about the pandemic, and perceived risk of infection. Similar to Rahman et al. (2023), this section is supplemental to KAP; however, we argue that these factors may influence the three dimensions of KAP and included them as independent variables in the subsequent analysis. The third section examines KAP related to COVID-19 personal protection and was devised with reference to the latest literature and policy documents (Cone Health, 2020; de Bruin and Bennett, 2020; Bagheri et al., 2021; Feldman et al., 2021; Nordsiek et al., 2021; Andrejko et al., 2022; Arefi et al., 2022; Chai et al., 2022; Eyre et al., 2022; Qalati et al., 2023; TravelHealthPro, 2023; WHO, 2023), and comprises a total of 33 items (16 knowledge, 7 attitude, 10 practice), with responses selected from “yes,” “no,” and “I don’t know.” In another similarity to Rahman et al. (2023), correct responses were assigned a value of 1, and incorrect responses and “I don’t know” were valued at 0, because “I don’t know” usually indicates that the person has suboptimal knowledge or does not know the answer (Luskin and Bullock, 2011). Detailed questions for the three sections of the questionnaire are presented in Sections “3.3 Personal protection knowledge for COVID-19,” “3.4 Personal protection attitudes regarding COVID-19,” and “3.5 Personal protection practices against COVID-19.”

Prior to data collection, the first author recruited a small group of volunteers (covering all age groups) on their own social media (WeChat) to complete the questionnaire and suggest improvements; these data were not included in subsequent analysis. To measure the reliability of the three KAP dimensions in the final version of the questionnaire, the Kuder-Richardson Formula 20 (KR-20) coefficient was calculated for each dimension, wherein the measure was considered reliable if the internal consistency value was greater than 0.70 (Sentandreu-Mañó et al., 2021). Cronbach’s alpha coefficients were not adopted because the responses were identified as “correct” and “incorrect” as a binary variable, and the KR-20 coefficient is more appropriate for dealing with this type of variable. The formula for calculating the KR-20 coefficient is as follows (**Equation 2**):


(2) KR-20=(k/(k-1))*(1-Σpqj/jσ)2

where:

k: Total number of items;

pj: Proportion of individuals who answered item j correctly;

qj: Proportion of individuals who answered item j incorrectly;

σ2: Variance of scores for all individuals.

The results showed high internal consistency for the three dimensions, with KR-20 coefficients of 0.779 (knowledge), 0.721 (attitude), and 0.897 (practice).

### 2.4 Data analysis

Microsoft Excel and IBM SPSS Statistics for Windows, Version 20.0 (Armonk, NY; IBM Corp) were used to compile and statistically analyze the questionnaire data, with the significance level at *p* < 0.05. Where appropriate, descriptive statistics were calculated. The means of the 900 sample scores on each of the three KAP dimensions (Equations 3–5) were calculated.


(3) $$A\,v\,e\,r\,a\,g\,e\,K\,n\,o\,w\,l\,e\,d\,g\,e=\frac{\Sigma_{1}^{n}\,k_{i}}{n}$$

(4) $$A\,v\,e\,r\,a\,g\,e\,A\,t\,t\,i\,t\,u\,d\,e=\frac{\Sigma_{1}^{n}\,A\,i}{n}$$

(5) $$A\,v\,e\,r\,a\,g\,e\,P\,r\,a\,c\,t\,i\,c\,e=\frac{\Sigma_{1}^{n}\,P_{i}}{n}$$

Where *n* = sample number;

The K_i_, A_i_, and P_i_ are the total scores of the ith sample for the knowledge, attitude, and practice dimensions, respectively.

Furthermore, an ordinal generalized linear model (OGLM) was implemented using SPSS to analyze the influence of sociodemographic characteristics and individual status on the KAP dimensions, wherein the former two items were the independent variables and the latter, the dependent variables. OGLM was chosen for its interpretable model parameters that describe the relationship between predictors and the ordinal response, and fit heterogeneous choice and related models better than ordered logit models that often violate the assumption of proportional odds (Zhang et al., 2021). In addition, OGLM is robust to deviations from normality and can handle skewed or non-normal data distributions, commonly encountered in real-world scenarios, thereby maximizing the use of available data by utilizing the full ordinal nature of the response variable. It avoids collapsing ordinal data into binary outcomes that may result in loss of information (Lyles et al., 2007), such as loss of granularity, variability, and statistical power. All results were reported as odds ratios (OR) and 95% confidence intervals (95% CI). The ggplot2 package of the R program (version 4.3.0) was used to make forest plots.

## 3 Results and discussion

### 3.1 Sample profile

Table 1 presents the demographic information of the participants. The sample was predominantly female (*n* = 546, 60.7%) and married (*n* = 564, 62.7%). More than half of the respondents were young (*n* = 312, 34.7%) and middle-aged (*n* = 360, 40%). Over 90% of participants lived in urban areas (*n* = 822, 91.3%). Only 15 participants reported that they had received only basic education. Most were retired (*n* = 207, 23%) or employed in the private sector (*n* = 162, 18%). The largest proportion were from the low-income group (*n* = 309, 34.3%), followed by those having average income (*n* = 300, 33.3%).

**TABLE 1:** Participants’ demographic information.

Items	Categories	Number	Percentage (%)
Gender	Male	354	39.3
	Female	546	60.7
Age	18–30 (youth)	312	34.7
	31–40 (young adult)	135	15.0
	41–60 (middle age)	360	40.0
	>60 (old age)	93	10.3
Marital status	Married	564	62.7
	Unmarried or single	336	37.3
Location	Rural area or suburban	78	8.7
	Urban area	822	91.3
Education	Primary	15	1.7
	Secondary	309	34.3
	Undergraduate/college	321	35.7
	Graduate	255	28.3
Occupation	Unemployed	117	13.0
	Student	153	17.0
	Retired	207	23.0
	Self-employed	60	6.7
	Private sector	162	18.0
	Public sector	138	15.3
	Government sector	63	7.0
Monthly income (RMB)	< 3,000 (low income)	309	34.3
	3,000–6,000 (average income)	300	33.3
	6,000-15,000 (above average income)	243	27.0
	>15,000 (high income)	48	5.3

### 3.2 Individual status regarding the COVID-19 pandemic

**FIGURE 4:**
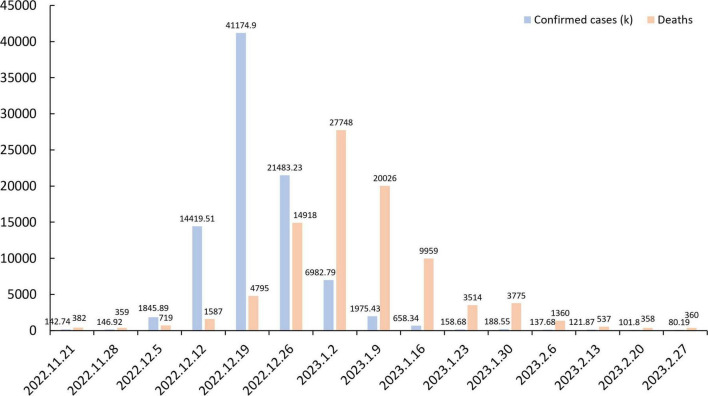
The number of confirmed infection cases and deaths reported from November 21, 2022 to February 27, 2023 in China (data resource: World Health Organization, 2023).

As shown in Table 2, 83.7% of respondents reported having been infected with COVID-19, and 95% had family or friends who were infected, corroborating recently reported trends. The number of infections increased significantly after China ceased strict control and quarantine measures in December 2022. From 3 January 2020 to 17 May 2023, at 5:43 pm CEST, China reported 99,256,991 confirmed COVID-19 cases to the WHO, with a total of 77,077,630 confirmed cases nationwide from 12 to 26 December 2022, accounting for 77.65% of the total reported cases (World Health Organization, 2023; Figure 4). Zhong Nanshan, a leading Chinese respiratory physician, said the December outbreak infected at least 85% of the population (Ye, 2023). Our questionnaire was conducted in April, at the point in time when all respondents had already experienced the heaviest wave of the pandemic, which may be a potential reason why respondents reported such a high rate of infection.

**TABLE 2:** Individual status regarding the COVID-19 pandemic.

Items	Responses	Number	Percentage (%)
1. Have you been infected with COVID-19?	Yes	753	83.7
	No	147	16.3
2. Have your family or friends been infected with COVID-19?	Yes	855	95.0
	No	21	2.3
	I don’t know	24	2.7
3. To what extent have you paid attention to COVID-19 recently?	Not concerned at all	276	30.7
	Somewhat concerned	552	61.3
	Greatly concerned	72	8.0
4. At what level do you think the area you live in is currently threatened by the pandemic?	Low risk	699	77.7
	Moderate risk	195	21.7
	High risk	6	0.7
5. Have you received any relevant training or education on COVID-19 prevention?	Yes	501	55.7
	No	399	44.3
6. Where did you learn about COVID-19 prevention?^a^	National and local authority	603	67.0
	Internet Media	708	78.7
	TV and radio	489	54.3
	Family or friends	507	56.3
	Poster	108	12.0
	Others	54	6.0
7. How would you rate your knowledge regarding COVID-19 prevention	Not at all	48	5.3
	Fairly well informed	765	85.0
	Very well informed	87	9.7

^a^Multiple answers could be selected.

Approximately 1/3 of respondents reported no concern at all about COVID-19 at present, 61.3% were somewhat concerned, and only 8% reported a high level of concern. Additionally, more than 70% considered the current pandemic threat to be very low, with only six participants (0.7%) considering it a high risk. This may be due to the fact that the survey was conducted in April 2023, when the pandemic has been fully controlled and cases of infection have decreased significantly. Thus, the low-risk perception of the public has led to reduced concern about COVID-19. Furthermore, individuals may not recognize the threats they face, and the low-risk perception stems from trust in scientific experts and authorities, and confidence in protective measures (Wachinger et al., 2013). However, this trust may lead people in areas lacking in protective measures tend to these natural risks, thus reducing their willingness and preparedness to act (Botzen et al., 2009). Therefore, despite the removal of strict regulatory measures, it is still recommended that authorities continue to engage and inform the public regarding personal NPI to avoid neglecting or underestimating the potential threat of the current pandemic. In terms of education on COVID-19 personal protection, more than half of the respondents indicated that they had received relevant training and 85% of the sample self-assessed that they were knowledgeable about personal protection, mostly from national and local authorities (67.0%), and Internet media (78.7%). This is consistent with previous findings in China, where the Internet was the most common source of information accessed by the public for COVID-19 knowledge (Wang H. et al., 2021). Moreover, the promotion of COVID-19 knowledge by the authorities also plays an important role, since the Chengdu city government, after December 2020, took great effort in promoting and educating people regarding COVID-19, specifically by organizing science education lectures, and showing promotional videos at community and street levels (The People’s Government of Sichuan Province, 2020). In the future, this initiative should continue and extend to COVID-19 personal protection, to reduce the potential risk.

### 3.3 Personal protection knowledge for COVID-19

**FIGURE 5:**
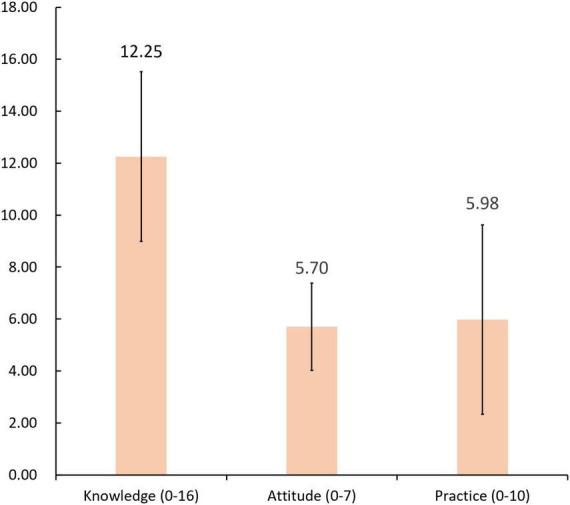
Mean (± standard deviation) of knowledge, attitude, and practice sections.

Responses regarding COVID-19 personal protection knowledge were collated (Table 3), and the mean score for this dimension was calculated (Figure 5). Overall, although a high standard of knowledge was not achieved (approximately 80% of questions were answered correctly; Rahman et al., 2023), the average of 76.6% correct (*M* = 12.25 ± 3.27) represents that participants have considerable knowledge related to COVID-19 personal protection. For example, the majority clearly knew how to wear masks properly (96%), to wear masks when entering public places (89.7%), to wash their hands regularly (89.3%), wash their hands after touching objects outdoors (83.3%), reduce the use of public transportation (82.7%), disinfect items at home regularly (88.3%), and wash their hands before rubbing their eyes and eating (90.3%). However, misunderstandings and uncertainties remain regarding some issues. For example, only half of the sample knew that they could get infected even while wearing a mask properly (49.7%), because masks merely reduce the risk of infection (Andrejko et al., 2022), and also because COVID-19 can enter the eye through respiratory droplets (Qalati et al., 2023; WHO, 2023). A significant proportion of respondents were also uncertain about the vaccine; for example, 40.6% did not know whether they could still get infected after vaccination, and 25% were unsure whether vaccinations allow a person to be in public without wearing a mask. Studies have shown that vaccines are not effective in the long term because of the emergence of viral variants (Jacobs et al., 2023), and that there is still a risk of contracting COVID-19 and transmitting it to others after vaccination; thus, WHO recommends that every effort should be made to maintain NPI even after vaccination (WHO, 2022). When it comes to wearing a mask in public, more than 70% of the respondents understood that they should ensure that they wear a mask regardless of the situation (e.g., length of stay, whether others are wearing masks or not). However, only 52.6% knew that outdoor gatherings are safer than indoor ones. Studies have shown that COVID-19 is an airborne disease that can be transmitted through particles (also known as aerosols) exhaled by an infected person that remain suspended in the air for a period rather than immediately falling to the ground, thereby increasing the probability of infection with increased infectious aerosol concentrations (Nordsiek et al., 2021). Thus, outdoor gatherings provide a lower infection risk.

**TABLE 3:** Responses toward knowledge of personal protection for COVID-19.

Items	Responses	Number (%)	Sources
1. The correct way to wear a mask is to make sure it covers your nose, mouth, and chin.	**Yes**	864 (96.0)	WHO, 2023
	No	21 (2.3)	
	I don’t know	15 (1.7)	
2. If the mask is worn correctly, you will not get COVID-19.	Yes	105 (11.7)	Andrejko et al., 2022; Qalati et al., 2023; WHO, 2023
	**No**	447 (49.7)	
	I don’t know	348 (38.6)	
3. You cannot be infected if you are vaccinated.	Yes	42 (4.7)	Eyre et al., 2022; WHO, 2022
	**No**	492 (54.7)	
	I don’t know	366 (40.6)	
4. A mask should be worn properly when entering public places to reduce the risk of infection.	**Yes**	807 (89.7)	Chai et al., 2022; WHO, 2023
	No	21 (2.3)	
	I don’t know	72 (8.0)	
5. Hands should be washed regularly and thoroughly with alcohol-based hand rub or soap and water to reduce the risk of infection.	**Yes**	804 (89.3)	WHO, 2023
	No	33 (3.7)	
	I don’t know	63 (7.0)	
6. If the mask is worn correctly, there is no risk of infection even when in contact with others.	Yes	108 (12.0)	Chai et al., 2022; WHO, 2023
	**No**	498 (55.3)	
	I don’t know	294 (32.7)	
7. If you touch anything outdoors, you should clean your hands thoroughly and immediately with alcohol-based hand rub or soap and water to reduce the risk of infection.	**Yes**	750 (83.3)	Feldman et al., 2021; WHO, 2023
	No	54 (6.0)	
	I don’t know	96 (10.7)	
8. When it is not possible to maintain a physical distance of at least 1 meter from others, or in poorly ventilated settings, it is necessary to wear a mask properly to reduce the risk.	**Yes**	801 (89.0)	Qalati et al., 2023; WHO, 2023
	No	21 (2.3)	
	I don’t know	78 (8.7)	
9. Reducing the usage of public transportation (e.g., buses, subways) can reduce the risk of infection.	**Yes**	744 (82.7)	Chai et al., 2022; Qalati et al., 2023; WHO, 2023
	No	24 (2.7)	
	I don’t know	132 (14.6)	
10. Hands need to be cleaned before you put your mask on, before and after you take it off, and after you touch it at any time.	**Yes**	774 (86.0)	WHO, 2023
	No	33 (3.7)	
	I don’t know	93 (10.3)	
11. Door handles, faucets, and phones in your home should be cleaned and disinfected regularly.	**Yes**	795 (88.3)	WHO, 2023
	No	51 (5.7)	
	I don’t know	54 (6.0)	
12. If vaccinated, it is safe to be in public without wearing a mask.	Yes	45 (5.0)	Chai et al., 2022; Qalati et al., 2023; WHO, 2023
	**No**	630 (70.0)	
	I don’t know	225 (25.0)	
13. Cleaning your hands immediately before rubbing your eyes and before eating reduces the risk of infection.	**Yes**	813 (90.3)	Qalati et al., 2023
	No	27 (3.0)	
	I don’t know	60 (6.7)	
14. For short stays in enclosed public places, it is safe not to wear a mask.	Yes	114 (12.7)	Qalati et al., 2023; WHO, 2023
	**No**	642 (71.3)	
	I don’t know	144 (16.0)	
15. In a public place, if everyone else is wearing a mask, it’s okay for me to not wear one.	Yes	66 (7.3)	Bagheri et al., 2021; WHO, 2023
	**No**	690 (76.7)	
	I don’t know	144 (16.0)	
16. Outdoor gatherings are safer (lower likelihood of infection) than indoor gatherings.	**Yes**	474 (52.6)	Nordsiek et al., 2021; WHO, 2023
	No	213 (23.7)	
	I don’t know	213 (23.7)	

Bold indicates the correct answer.

**FIGURE 6:**
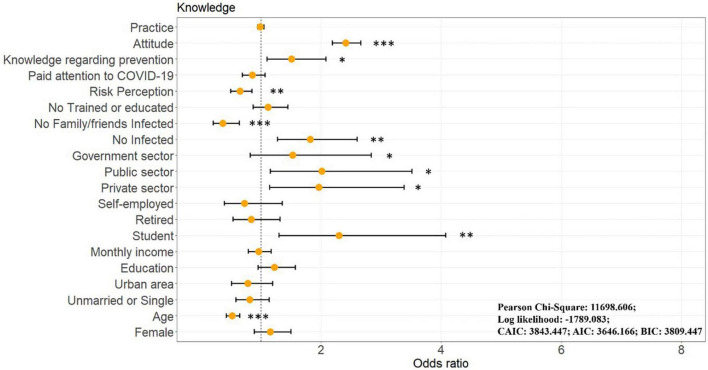
Results of ordinal generalized linear model for COVID-19 personal protection knowledge. *p < 0.05, **p < 0.01, ***p < 0.001.

To examine the demographic characteristics and individual status that influence knowledge, OGLM was applied (Figure 6 and Supplementary Appendix Table 1). Regarding demographic characteristics, older (OR = 0.517 [0.418, 0.640], *p* < 0.001) respondents possessed less correct knowledge. The result is inconsistent with those of previous studies; Cotten and Gupta (2004) found that older people have more health needs than younger people and are, therefore, more likely to seek and find accurate health information, with studies in Saudi Arabia, Indonesia, and Malaysia supporting this conclusion (Azlan et al., 2020; Alnasser et al., 2021; Muslih et al., 2021). However, our findings suggest that younger populations are more likely to know about proper protection in the post-pandemic era, which is reasonable because previous studies have generally been conducted in the pre-pandemic and mid-pandemic periods, where there was a strong motivation to learn about protection due to higher mortality risk in middle-aged and older populations (Ioannidis et al., 2020), however, with the increasing number of infections across all age groups, it is likely that younger populations have also learned from the pandemic. In addition, younger populations are often more adept at using the Internet and have access to more accurate knowledge (Abdel Wahed et al., 2020). Furthermore, compared to the unemployed, retired, and self-employed groups, students (OR = 2.299 [1.298, 4.070], *p* = 0.004), private sector (OR = 1.961 [1.138, 3.380], *p* = 0.015), public sector (OR = 2.015 [1.156, 3.514], *p* = 0.013), and government employees (OR = 1.524 [0.819, 2.836], *p* = 0.041) held more accurate knowledge. The type of employment, usually related to socioeconomic status, potentially influences the level of knowledge acquired (Sujarwoto et al., 2022); thus, the retired, unemployed and non-stable employed groups were least knowledgeable among the respondents. The university students have a higher level of education, are usually young and, therefore, more likely to have accurate knowledge (Abdel Wahed et al., 2020). In terms of individual status, those who had not been previously infected with COVID-19 (OR = 1.819 [1.273, 2.600], *p* = 0.001), their family/friends got infected (OR = 0.361 [0.204, 0.637], *p* < 0.001), assessed themselves as having more knowledge of COVID-19 personal protection (OR = 1.510 [1.096, 2.081], *p* = 0.012), and perceived lower risk (OR = 0.647 [0.492, 0.850], *p* = 0.002) had more accurate knowledge. These results are reasonable since, because of their greater knowledge of personal protection, these respondents were not infected. Further, the results suggest that the self-assessment is consistent with the actual measurement, demonstrating that the items on the scale are useful knowledge that residents apply to their daily lives. Reporting that a family member had been infected is a reasonable result that led to higher levels of knowledge among respondents, and there are two potential reasons for this: (1) infection of a family member leads to a heightened sense of protection and vigilance among other members, which leads to stronger motivation to learn about personal protection; and (2) during the pandemic in China, if a family member becomes infected, it is often necessary to have other healthy family members to care for them (provide food, water, and medicines), and thus this requires those healthy family members to be more attentive to their personal protection. Also, higher subjective knowledge is associated with lower perceived risk (Jaccard et al., 2005), as individuals who perceive themselves to be well informed may believe that their knowledge counteracts hazards, thus reducing subjective perceptions of risk (Buratti and Allwood, 2019). Finally, respondents with higher attitude scores they also had higher levels of knowledge, which is consistent with the findings of previous studies that attitude is closely related to knowledge, as positive attitudes promote individuals to learn more relevant knowledge (Rahman et al., 2023).

### 3.4 Personal protection attitudes regarding COVID-19

The responses of personal protection attitudes are shown in Table 4. Compared to knowledge and practice, the participants surveyed in this study had highly positive attitudes toward personal protective behavior, *M* = 5.70 ± 1.68 (mean correct rate 81.4%; Figure 5). Specifically, 85.3% agreed that they should spread knowledge about personal protective measures to their family and friends, 90% believed that the level of pandemic risk at the destination should be carefully considered before traveling, 83% supported activities or training on COVID-19 prevention in their community, 92.7% agreed that proper mask wearing and regular hand washing are effective in preventing transmission, and 80% agreed that physical distance should be maintained in public places. However, only 63% of participants insisted on wearing masks in public when they felt uncomfortable. Leung et al. (2020) show that one of the most effective preventive behaviors is wearing a mask that is effective against airborne transmission of viruses; however, the discomfort factor (e.g., thermal sensation) may still reduce people’s willingness to wear them (Milošević et al., 2022). Therefore, the development of thermally regulated masks and respirators is important to encouraging daily mask-wearing behavior in the post-pandemic era (O’Dowd et al., 2020), and new comfortable mask materials may greatly reduce the probability of removing masks in public places. Furthermore, only 76.3% agreed that unnecessary gatherings and visits to public places should be avoided as much as possible. Although strict movement restrictions have been removed, there is a potential risk of disease transmission wherever large numbers gather. Therefore, unnecessary excursions to public places, such as dining out and store visits, should be avoided as far as possible.

**TABLE 4:** Responses regarding attitudes toward personal protection against COVID-19.

Items	Responses	Number (%)	Sources
1. I should inform my family and friends about personal protection against COVID-19.	**Yes**	768 (85.3)	de Bruin and Bennett, 2020; Arefi et al., 2022
	No	33 (3.7)	
	I don’t know	99 (11.0)	
2. I should carefully consider the level of infection risk at the destination before making travel or trip plans.	**Yes**	810 (90.0)	TravelHealthPro, 2023
	No	39 (4.3)	
	I don’t know	51 (5.7)	
3. I support activities or training regarding COVID-19 prevention in the community where I live.	**Yes**	747 (83.0)	de Bruin and Bennett, 2020; Arefi et al., 2022
	No	60 (6.7)	
	I don’t know	93 (10.3)	
4. I agree that proper mask wearing and regular hand washing can be effective in preventing the spread of COVID-19.	**Yes**	834 (92.7)	WHO, 2023
	No	18 (2.0)	
	I don’t know	48 (5.3)	
5. I can take off my mask at any time in public if I feel uncomfortable.	Yes	177 (19.7)	WHO, 2023
	**No**	567 (63.0)	
	I don’t know	156 (17.3)	
6. I think everyone should try to avoid unnecessarily remaining and gathering in public places.	**Yes**	687 (76.3)	Cone Health, 2020
	No	90 (10.0)	
	I don’t know	123 (13.7)	
7. I agree that it is necessary to maintain a physical distance of at least 1 meter from others in public places.	**Yes**	720 (80.0)	WHO, 2023
	No	72 (8.0)	
	I don’t know	108 (12.0)	

Bold indicates the correct answer.

**FIGURE 7:**
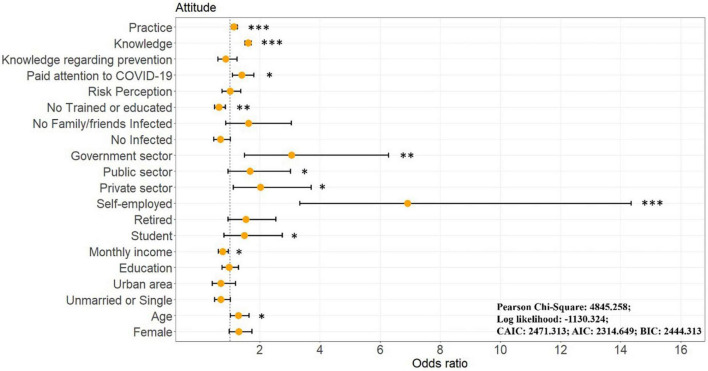
Results of ordinal generalized linear model for attitudes toward COVID-19 personal protection. *p < 0.05, **p < 0.01, ***p < 0.001.

OGLM was applied to examine whether demographic characteristics and individual states influenced attitudes (Figure 7 and Supplementary Appendix Table 1). Those who was older (OR = 1.288 [1.015, 1.635], *p* = 0.038), and had a low income (OR = 0.762 [0.616, 0.944], *p* = 0.013) had more positive attitudes. First, unlike the knowledge outcome that more correct knowledge among younger people, the older age groups had more positive attitudes toward COVID-19 personal protection. Although, young people can have more access to correct knowledge, the older people, as a vulnerable group in a pandemic, have more health needs than younger groups, which leads to a higher likelihood of higher positive attitudes (Cotten and Gupta, 2004). Besides, in line with the results of a previous study (Sujarwoto et al., 2022), low-income populations are more likely to have more positive attitudes, due to the fact that low-income groups have less ability to afford healthcare and therefore show stronger positive attitudes toward protecting their own and their family’s health, as well as a greater willingness to learn and share relevant information about COVID-19 personal protection. Further, compared to the unemployed population, students (OR = 1.483 [0.801, 2.743], *p* = 0.036), self-employed (OR = 6.906 [3.325, 14.345], *p* < 0.001), private sector (OR = 2.026 [1.110, 3.698], *p* = 0.021), public sector (OR = 1.678 [0.932, 3.021], *p* = 0.034), and government employees (OR = 3.051 [1.482, 6.283], *p* = 0.002) had more positive attitudes. Since students and employed persons are more likely to be in frequent contact with other people (passively or actively, sometimes by necessity), the need for knowledge and positive attitudes toward personal protection is more strongly experienced. In terms of individual status, those who received training (OR = 0.642 [0.485, 0.852], *p* = 0.002) and were more concerned about the COVID-19 pandemic (OR = 1.394 [1.084, 1.791], *p* = 0.010) had stronger positive attitudes. The Chengdu government considers community units to be an important gateway for outbreak prevention and control and has, therefore, has set up a “micro-network” of grassroots governance mechanisms, with family doctors and counselors in each community unit, and regular community education and training (Sichuan View News, 2023). This supports previous research showing that disaster management training can be effective in enhancing positive attitudes toward disaster response behavior (Mirzaei et al., 2019). Moreover, increased attention to disasters has been found to positively influence attitudes toward the event; greater (and easier) access to news that allows the public to be well informed about the details of a disaster, likely enhances their positive attitude (Zhu and Zhang, 2017). Finally, respondents with higher knowledge scores were more likely to have positive attitudes (OR = 1.608 [1.523, 1.698], *p* < 0.001), which is consistent with finding as for whether or not they received training. In addition, this result demonstrates the role that knowledge plays in shaping attitudes, i.e., knowledge can lead to more positive attitudes toward COVID-19 protection.

### 3.5 Personal protection practices against COVID-19

The responses of personal protection practices are shown in Table 5. Despite the high level of positive attitudes and the acceptable level of knowledge, the current average of 59.8% (*M* = 5.98 ± 3.65) correct responses indicates that practical behavior is inadequate in the surveyed sample (Figure 5). First, only half of the respondents indicated that they would avoid crowded public places (outdoors: 54.7%; indoors: 56.3%). As mentioned earlier, because masks and vaccines provide only limited protection, crowded places are more likely to spread infection (both indoors and outdoors). Therefore, proper practice entails avoid unnecessary visits to these crowded public places to reduce the risk of infection. Second, less than 60% of the respondents reported that they try to maintain more than 1 meter of physical distance from others (outdoors: 56.7%; indoors: 53.7%). A prior meta-analysis of 172 observational studies indicated that virus transmission was lower at physical distances of 1 m or greater, with protection increasing with distance (Chu et al., 2020). Finally, approximately 53% of respondents said that they would ensure they wore a mask when talking to others (outdoors: 53.7%; indoors: 53.3%). A systematic study showed that various exhalation activities can produce respiratory droplets and aerosols that, when produced by infected individuals, may contain infectious viruses. Additional precautions must be taken to reduce short- and long-range droplet/aerosol transmission, particularly ventilation, air filtration, and mask fit, to prevent future outbreaks (Wang C. C. et al., 2021).

**TABLE 5:** Responses regarding the practice of personal protection against COVID-19.

Items	Responses	Number (%)	Sources
1. In the recent week, I wore my mask properly to reduce the risk of infection when I was in the outdoor public places (e.g., parks, squares).	**Yes**	540 (60.0)	WHO, 2023
	No	237 (26.3)	
	I am not sure	123 (13.7)	
2. In the recent week, I washed my hands regularly even when I was in outdoor public places.	**Yes**	675 (75.0)	WHO, 2023
	No	138 (15.3)	
	I am not sure	87 (9.7)	
3. In the recent week, I tried to avoid going to outdoor public places where many people are present.	**Yes**	492 (54.7)	Qalati et al., 2023; WHO, 2023
	No	285 (31.7)	
	I am not sure	123 (13.6)	
4. In the recent week, I tried to keep a physical distance of at least 1 meter when meeting with others at outdoor public places.	**Yes**	510 (56.7)	Chai et al., 2022; Qalati et al., 2023; WHO, 2023
	No	255 (28.3)	
	I am not sure	135 (15.0)	
5. In the recent week, I always made sure to wear my mask properly when talking to others at outdoor public places.	**Yes**	483 (53.7)	WHO, 2023
	No	288 (32.0)	
	I am not sure	129 (14.3)	
6. In the recent week, I wore my mask properly to reduce the risk of infection when I was in the indoor public places (e.g., stores, shopping malls).	**Yes**	549 (61.0)	WHO, 2023
	No	243 (27.0)	
	I am not sure	108 (12.0)	
7. In the recent week, I washed my hands regularly when I was in indoor public places.	**Yes**	660 (73.3)	WHO, 2023
	No	165 (18.3)	
	I am not sure	75 (8.3)	
8. In the recent week, I tried to avoid going to indoor public places where many people are present.	**Yes**	507 (56.3)	Qalati et al., 2023; WHO, 2023
	No	267 (29.7)	
	I am not sure	126 (14.0)	
9. In the recent week, I tried to keep a physical distance of at least 1 meter when meeting with others in indoor public places.	**Yes**	483 (53.7)	Chai et al., 2022; Qalati et al., 2023; WHO, 2023
	No	282 (31.3)	
	I am not sure	135 (15.0)	
10. In the recent week, I always made sure to wear my mask properly when talking to others at indoor public places.	**Yes**	480 (53.3)	WHO, 2023
	No	273 (30.3)	
	I am not sure	147 (16.3)	

Bold indicates the correct answer.

**FIGURE 8:**
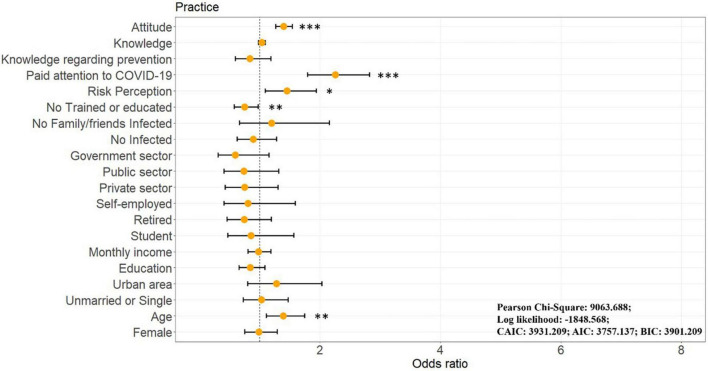
Results of ordinal generalized linear model for COVID-19 personal protection practice. *p < 0.05, **p < 0.01, ***p < 0.001.

Figure 8 and Supplementary Appendix Table 1 show the OGLM results for demographic characteristics and individual status that influence overall practice behavior. Unexpectedly, no demographic characteristics other than age (OR = 1.390 [1.109, 1.744], *p* = 0.004) significantly influenced people’s practices (*p* > 0.05). In terms of personal status, those with training (OR = 0.746 [0.573, 0.970], *p* = 0.029), higher COVID-19 risk perception (OR = 1.454 [1.091, 1.938], *p* = 0.011), and those who were more concerned about the COVID-19 pandemic (OR = 2.252 [1.797, 2.824], *p* < 0.001) scored higher in overall practice. In addition, people with positive attitudes are more likely to follow personal protective practice behaviors in their daily lives (OR = 1.395 [1.264, 1.540], *p* < 0.001).

**FIGURE 9:**
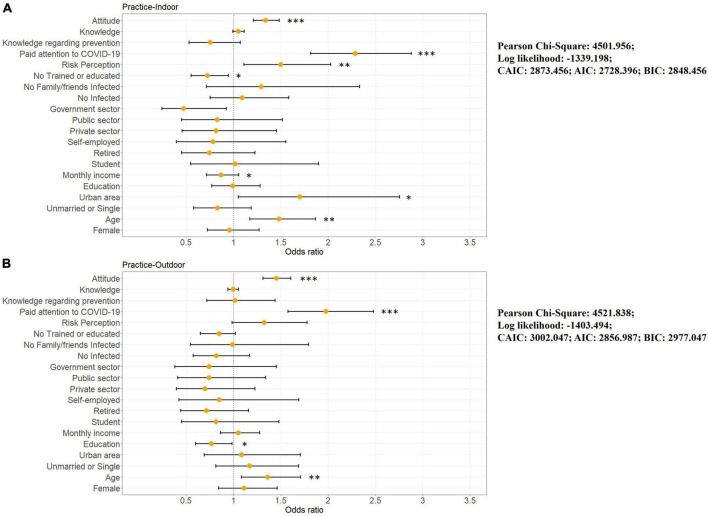
Results of ordinal generalized linear model for COVID-19 personal protection indoor and outdoor practice; **(A)** indoor practice, **(B)** outdoor practice. *p < 0.05, **p < 0.01, ***p < 0.001.

To examine whether there were differences in practice behaviors between different populations indoors (e.g., stores, shopping malls, items 6–10) and outdoors (e.g., parks, squares, items 1–5), OGLM was executed twice, and the scores of indoor and outdoor practices were set as dependent variables (Figure 9 and Supplementary Appendix Table 2). Notably, when we divided overall practice behavior into indoor and outdoor, different sociodemographic characteristics and individual status significantly influenced both scores.

Specifically, in indoor environments, older (OR = 1.479 [1.171, 1.867], *p* = 0.001), living in the urban area (OR = 1.701 [1.050, 2.755], *p* = 0.031), and low-income participants (OR = 0.867 [0.714, 1.054], *p* = 0.038) showed better practice behavior. This may because the public recognizes the greater risk of infection in indoor public places and vulnerable populations (here the elderly, low-income populations) tend to follow personal protective behaviors more appropriately to reduce the risk of infection. In addition, respondents in urban environments scored higher on protective behaviors in indoor environments, which is due to the fact that for people living in urban areas are more likely to visit and use indoor public places (e.g., offices, stores, shopping malls), and therefore pay more attention to personal protection in indoor environments. On the other hand, according to previous reports given that the higher urban infection rates due to population density (Hamidi et al., 2020), and many infections occur indoors (Moritz et al., 2021), which potentially increases the alertness of indoor visitors. In outdoor settings, those with low education (OR = 0.766 [0.596, 0.985], *p* = 0.037), and older people (OR = 1.360 [1.083, 1.707], *p* = 0.008) performed better. The low-education population and the old performed better regarding outdoor settings, which may be explained as the same as in indoor settings. Educational status is usually correlated with income (Sujarwoto et al., 2022), and the lower-educated and the old population, as a vulnerable group, have less access to healthcare and are, therefore, more aware of personal protective behavior practice, even outdoors.

In terms of individual status, those with training (OR = 0.722 [0.551, 0.947], *p* = 0.019), more concerned about the pandemic (OR = 2.284 [1.814, 2.877], *p* < 0.001), and with higher risk perception (OR = 1.499 [1.109, 2.027], *p* = 0.009) scored higher on practice in indoor settings, whereas only those who were more concerned about the pandemic scored higher on practice in outdoor environments (OR = 1.976 [1.575, 2.480], *p* < 0.001). In addition, those who maintained positive attitudes toward COVID-19 personal protection were more likely to adhere to protective behaviors both indoors (OR = 1.338 [1.209, 1.482], *p* < 0.001) and outdoors (OR = 1.451 [1.311, 1.605], *p* < 0.001). As previously discussed, disaster management training can be effective in enhancing positive attitudes toward disaster response behaviors (Mirzaei et al., 2019) and greater attention to disasters also positively influences attitudes toward the event (Zhu and Zhang, 2017), and, finally, positive attitudes lead to higher practice outcomes (Sujarwoto et al., 2022). However, most of the variables did not have a significant effect on practice scores for outdoor environments, even the trained or with higher risk perception population did not show significant differences in practice behaviors in the outdoor environment, which probably because the surrounding population density is lower in most outdoor situations, causing the public to unconsciously relax their vigilance.

Overall, the vulnerable groups (like the elderly, low-income populations) are more aware of personal protective behaviors than non-vulnerable groups, indicating that the latter are more exposed to the risk of COVID-19. However, in general, non-vulnerable groups have easier access to healthcare, medications, and treatments than vulnerable groups, which will undoubtedly exacerbate the pressure on healthcare resources in the next outbreak, thus leading to the possibility of inequitable healthcare access. Additionally, outdoor public spaces (often low-population-density environments) potentially allow visitors to let their guard down and thus reduce the practice of protective behaviors, which affects even trained populations as well as those with a higher perceived risk level. Third, only those who maintained positive attitudes toward COVID-19 personal protection were more likely to maintain practice behaviors in both indoor and outdoor environments, and the amount of knowledge did not significantly contribute to respondents’ actual protection behaviors, unlike the previous emphasis on knowledge (see Bagheri et al., 2021; Andrejko et al., 2022; Arefi et al., 2022; Sujarwoto et al., 2022). These findings are important for developing future urban health policies.

### 3.6 Non-linear relationships among the three dimensions of KAP

**FIGURE 10:**
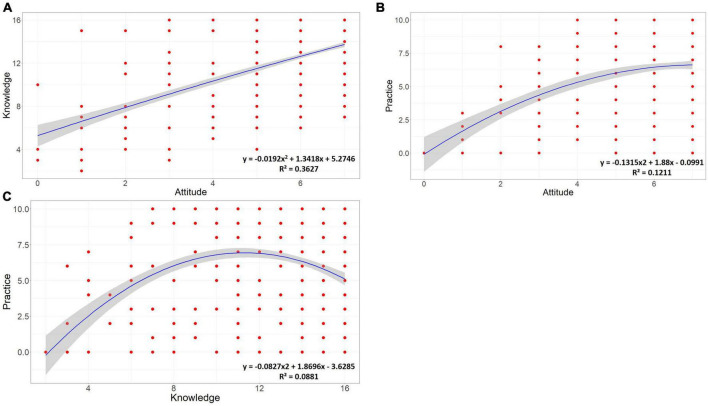
Relationships among the three KAP dimensions. **(A)** Attitude-knowledge; **(B)** attitude-practice; **(C)** knowledge-practice.

A polynomial regression by R program was executed to examine the nonlinear relationship between the three dimensions of KAP (Figure 10). Overall, respondents’ knowledge and practice behaviors increased with positive attitudes (Figures 10A, B). First, the public knowledge was positively correlated with attitudes, which is consistent with previous studies (Alnasser et al., 2021; Reuben et al., 2021; Sujarwoto et al., 2022). Then, practice scores increased with attitude scores, but the tendency to increase decreased when the attitudes scores increased beyond a point, indicating that individuals’ attitudes have limited incentives for actual behavior. Particularly, if people become aware that these practices may be unnecessary (e.g., it may be safe to not wear a mask outdoors if there is a low density of people around) or non-mandatory (the current Chinese government no longer requires citizens to strictly implement NPI measures), the propensity to practice may decrease, even if individuals have strong attitudes. Finally, it is worth noting that the relationship between knowledge and practice is an inverted U-shaped (Figure 10C), suggesting that while knowledge increases to a threshold (in this study, 68.75–75% of the correctness rate), the associated protective behavior then begins to decrease, which the result may explains why the relationship between knowledge and practice is insignificant in all OGLM models. Besides, this directly supports previous findings that as populations acquire more knowledge, they are more likely to overestimate the ability of the knowledge they possess to protect against risk and underestimate the negative effects of risk (Buratti and Allwood, 2019). This result is paradoxical for city managers because on the one hand they want the public to learn more to protect themselves against COVID-19 risks, but on the other hand too much knowledge may lead to complacency and reduce their actual protective behaviors. Therefore, we recommend that, in the future, managers should deliver education about personal protection from COVID-19 which emphasizes the importance of adopting protective behaviors and the potential risks of ignoring them, to increase the public’s positive attitude toward personal protection and their tendency to protect themselves.

### 3.7 Limitations

First, this study used a web-based questionnaire to collect data; therefore, there may be some bias in the sample distribution, such as a smaller proportion of older adults who likely less adept at using cell phones or computers. These biases may limit the external validity of this study and the extent to which the results can be applied to other settings or populations. In addition, people who were not interested or did not want to discuss the topic of this study may have been excluded. Second, cost constraints limited the data collection to the city level, and future studies could consider a deeper survey at the neighborhood or street level to provide a clear picture of the residents in different areas of the city. Third, this was a cross-sectional survey. City managers and policymakers may want to conduct more surveys at different time points as the new normal evolves. Also, comparative analyses with data from earlier time periods are meaningful, therefor more works need to do by comparing the data from this study with previous KAP data from the Chengdu area in the future. Moreover, studies from other cities and countries are required to monitor the extent of KAP in each region, which is important for global preparedness against the next pandemic. Fourth, although the most recent literature and policy documents were referenced to formulate the survey questions, as new technologies and conditions emerge, the current questionnaire would need to be accordingly adapted. Finally, this study innovatively included the individual status dimension, and these items were found to influence public KAP levels. therefore, Thus, more variables that may potentially influence public KAP levels need to be explored and discussed in the future.

## 4 Study implications and contributions

### 4.1 Potential international relevance and impacts

At the first, comparing the results of the current study with similar studies conducted in other parts of the world can provide insights into how different populations respond to and adopt COVID-19 protective behaviors. This can help identify trends and variations in knowledge, attitudes, and practices across cultures and regions. Second, according to the United Nations Sustainable Development Goals (SDGs), healthy populations are key to building resilient cities. The survey reveals knowledge gaps or negative attitudes toward certain personal protection behaviors, which can inform targeted educational activities or public health strategies in other region and countries. As such, this study is one of the key pieces of the puzzle for a global KAP survey in the post-pandemic era. Third, epidemiologists can use the data collected by this and other KAP surveys to refine epidemiologic models, which can help predict the spread of the virus and the impact of interventions in different settings.

### 4.2 Contributions for sustainability of urban cities regarding COVID-19/alike crisis control

More than half of the world’s population lives in cities, and the 11th of the UN Sustainable Development Goals aims to “make cities and human settlements inclusive, safe, resilient and sustainable” by 2030, the people therefore form the basis of sustainable cities. Espey (2019) stated that the Sustainable Development Goals without data will inevitably fail. A recent study from Japan has begun to discuss the progress of SDG 11 indicators in the local cities, yet the need for healthy populations or socio-ecological aspects of resilient cities is still not addressed (Yamasaki and Yamada, 2022). Thus, this study demonstrates that the KAP survey can be a reliable indicators data collection tool for COVID-19/alike crisis risk knowledge/protection behaviors. Second, humans will continue to coexist with the virus for a long time to come. Variations, diminishing vaccine efficacy, and the emergence of new infectious diseases (e.g., monkeypox, Matsee et al., 2023) demonstrate that NPI will be a more implementable and sustainable means of protection for future cities. Therefore, in the post-pandemic era, based on the related KAP surveys managers and policymakers could promote knowledge of personal prevention and encourage attitudes and practices that reduce the risk of COVID-19/alike crisis spreading; also, the KAP surveys can be used to assess the public’s strategies to protect themselves from specific disasters. These contributions are valuable for sustainable urban development regarding COVID-19/alike crisis control.

### 4.3 Control measures, solutions, and policy implications

First and foremost, in contrast to the previous emphasis on the importance of knowledge, our study found that positive attitudes toward personal protection are more important in the post-pandemic era, as this directly and significantly influences residents’ practice behaviors. Besides, despite the withdrawal of strict management measures, it is still recommended that authorities encourage and educate on personal NPI while emphasizing the importance of associated protective behaviors and the potential risks of ignoring them, thus enhancing positive attitudes toward personal protection and the propensity for practice to prevent the public from underestimating the potential threat of the current pandemic. Second, the potential casualties from a pandemic can be reduced through early intervention based on sociodemographic information. Using demographic data, infection risk assessment can focus on the populations most at-risk. Third, although WHO has lowered the risk level of COVID-19, humans must coexist with the pandemic for an extended period; thus, understanding changes in public KAP regarding personal protective behaviors in the post-pandemic era is important for the sustainability of cities and communities. In addition, this study reveals that the public’s personal protective behaviors in the post-pandemic era are inadequate, specifically maintaining physical distance, avoiding visits to crowded public places, and wearing masks. These findings can inform targeted education and encouragement policies by city managers. Fourth, the development and use of new mask materials that enhance comfort (e.g., decrease thermal sensation) can effectively reduce the probability of removing masks in public places, thereby reducing the risk of outbreaks. Fifth, ensuring that the public is informed about the pandemic from a variety of sources can enhance positive attitudes toward personal protection. Moreover, outdoor public spaces (squares, parks, and public green spaces) in cities may be potential transmission areas for the next outbreak due to the public lets its guard down. Therefore, focus on these areas needs to be strengthened to emphasize and advise on personal protective behaviors. In particular, persuasion of non-vulnerable populations to practice NPI could reduce the potential pressure on urban medical resources. Finally, the KAP survey can help identify potential risk areas in the city (such as the outdoor public spaces surveyed in this study), as well as high infect risk populations (such as the non-vulnerable groups in this study). The results of the study can therefore help city authorities to allocate resources such as medical supplies, testing facilities and public health activities more efficiently based on the specific knowledge, attitudes and practices of the city population.

## 5 Conclusion

The United Nations 2030 Agenda for Sustainable Development recognizes and reaffirms the urgent need for disaster risk reduction to promote the resilience and sustainability of cities and human settlements. With the advent of the post-pandemic era, national-level preparedness measures will be largely replaced by personal protective behaviors. Therefore, in the context of the new normal, encouraging the public to implement proper COVID-19 personal protection behaviors is of great significance for the sustainable development of cities and communities. To the best of our knowledge, there are few studies that address public protective behaviors in the post-pandemic era, and this baseline survey closes this knowledge gap, yielding many valuable findings: (1) approximately 1/3 of the respondents in this study indicated that they are currently not concerned about COVID-19 at all, which needs to be brought to the attention of the authorities because the pandemic is still not over and excessive public laxity could pose a great risk to urban public health; (2) more than half of the respondents indicated that they had received relevant training and 85% of the sample self-assessed as being knowledgeable about personal protection, indicating that the promotion and education (lectures and training) measures should be continued in the future; (3) although the participants possessed fairly accurate knowledge related to COVID-19 personal protection and had highly positive attitudes, their practice behaviors were inadequate; (4) knowledge scores were higher in younger and employed participants; (5) attitude scores were higher in older, low-income, and employed participants; (6) individual status dimensions (such as whether previously infected, trained, and concerned about the pandemic) were found to significantly influence the three KAP dimensions, making this data valuable; (7) respondents with different sociodemographic characteristics and individual statuses show significant differences in their indoor and outdoor safety practices; (8) disaster training and higher concern with regard to the pandemic positively influenced the public’s scores on COVID-19 attitudes toward personal protective behaviors, leading to higher practice behaviors; (9) vulnerable groups (older people, people with low education levels, and low-income groups) performed better in indoor/outdoor practices. Further, most people relaxed their vigilance in outdoor environments, thus reducing practice behaviors; (10) attitudes offer limited incentive for practice when people realize that practice behaviors may be unnecessary or non-mandatory; the propensity to practice may be low even when individuals have strong attitudes; (11) when knowledge increases to a threshold level (68.75–75% in this study), individuals’ protective behaviors start to decrease because they may overestimate the risk resistance potential of the knowledge they possess. Finally, a series of measures are also proposed based on these results and findings, as well as recommendations to help cities deal with potential future COVID-19 and alike crisis.

## Data availability statement

The original contributions presented in this study are included in this article/Supplementary material, further inquiries can be directed to the corresponding author.

## Author contributions

SL: Conceptualization, Data curation, Formal analysis, Funding acquisition, Investigation, Methodology, Software, Validation, Visualization, Writing – original draft, Writing – review & editing. JX: Conceptualization, Funding acquisition, Investigation, Project administration, Resources, Supervision, Validation, Writing – original draft, Writing – review & editing. JC: Investigation, Methodology, Visualization, Writing – review & editing. HL: Investigation, Writing – review & editing. SZ: Investigation, Writing – review & editing

## Footnotes

1
https://www.credamo.world/
