# Supplementary material for: Survey of public knowledge, attitudes, and practices regarding personal protection against COVID-19 in the post-pandemic era
Source: Front Psychol. 2024 Jun 10;15:1411055. doi: 10.3389/fpsyg.2024.1411055 (PMC11195805; doi:10.3389/fpsyg.2024.1411055)
Supplement: Supplementary file 1 [file Table_1.docx]

# Supplementary Material

Table A1. OGLM results for the three KAP dimensions of different population groups

| **Variables** |  | Exp(B) (95% Confidence interval) | | |
| --- | --- | --- | --- | --- |
|  | **Items** | **Knowledge** | **Attitude** | **Practice** |
| **Sociodemographic characteristics** | **Gender (=Female)** | 1.154 (0.888, 1.498) | 1.300 (0.972, 1.738) | 0.983 (0.749, 1.289) |
|  | **Age** | 0.517 (0.418, 0.640) *** | 1.288 (1.015, 1.635) * | 1.390 (1.109, 1.744) ** |
|  | **Marital status (=Unmarried or Single)** | 0.812 (0.581, 1.134) | 0.702 (0.485, 1.017) | 1.029 (0.721, 1.468) |
|  | **Location (=Urban area)** | 0.778 (0.508, 1.190) | 0.697 (0.412, 1.181) | 1.275 (0.800, 2.030) |
|  | **Education** | 1.221 (0.950, 1.570) | 0.978 (0.742, 1.287) | 0.844 (0.658, 1.082) |
|  | **Monthly income** | 0.959 (0.788, 1.168) | 0.762 (0.616, 0.944) * | 0.977 (0.806, 1.183) |
|  | **Occupation** | *Reference group - Unemployed* | | |
|  | Student | 2.299 (1.298, 4.070) ** | 1.483 (0.801, 2.743) * | 0.856 (0.468, 1.562) |
|  | Retired | 0.834 (0.529, 1.315) | 1.539 (0.937, 2.527) | 0.740 (0.459, 1.192) |
|  | Self-employed | 0.725 (0.389, 1.350) | 6.906 (3.325, 14.345) *** | 0.805 (0.409, 1.586) |
|  | Private sector | 1.961 (1.138, 3.380) * | 2.026 (1.110, 3.698) * | 0.746 (0.427, 1.301) |
|  | Public sector | 2.015 (1.156, 3.514) * | 1.678 (0.932, 3.021) * | 0.734 (0.409, 1.318) |
|  | Government sector | 1.524 (0.819, 2.836) * | 3.051 (1.482, 6.283) ** | 0.594 (0.305, 1.155) |
| **Individual status** | **Infected (=No)** | 1.819 (1.273, 2.600) ** | 0.685 (0.466, 1.006) | 0.893 (0.625, 1.276) |
|  | **Family/friends Infected (=No)^a^** | 0.361 (0.204, 0.637) *** | 1.623 (0.866, 3.041) | 1.195 (0.663, 2.154) |
|  | **Trained or educated (=No)** | 1.118 (0.866, 1.445) | 0.642 (0.485, 0.852) ** | 0.746 (0.573, 0.970) ** |
|  | **Risk Perception** | 0.647 (0.492, 0.850) ** | 1.006 (0.743, 1.362) | 1.454 (1.091, 1.938) * |
|  | **Concerned about COVID-19** | 0.853 (0.684, 1.064) | 1.394 (1.084, 1.791) * | 2.252 (1.797, 2.824) *** |
|  | **Knowledge regarding prevention** | 1.510 (1.096, 2.081) * | 0.867 (0.607, 1.239) | 0.839 (0.596, 1.182) |
| **KAP** | **Knowledge** | - | 1.608 (1.523, 1.698) *** | 1.032 (0.977, 1.089) |
|  | **Attitude** | 2.410 (2.186, 2.657) *** | - | 1.395 (1.264, 1.540) *** |
|  | **Practice** | 0.985 (0.949, 1.022) | 1.133 (1.089, 1.180) *** | - |
| **Pearson Chi-Square** |  | 11698.606 | 4845.258 | 9063.688 |
| **Log likelihood** |  | -1789.083 | -1130.324 | -1848.568 |
| **CAIC** |  | 3843.447 | 2471.313 | 3931.209 |
| **AIC** |  | 3646.166 | 2314.649 | 3757.137 |
| **BIC** |  | 3809.447 | 2444.313 | 3901.209 |

Note: AIC, Akaike information criterion; BIC, Bayesian information criterion; CAIC, Consistent Akaike information criterion.

a Eliminated “I don’t know” responses.

* p < 0.05, ** p < 0.01, *** p < 0.001.
