# Supplementary material for: Survey of public knowledge, attitudes, and practices regarding personal protection against COVID-19 in the post-pandemic era
Source: Front Psychol. 2024 Jun 10;15:1411055. doi: 10.3389/fpsyg.2024.1411055 (PMC11195805; doi:10.3389/fpsyg.2024.1411055)
Supplement: Supplementary file 2 [file Table_2.docx]

# Supplementary Material

Table A2. OGLM results regarding practices (indoor and outdoor) of different population groups

| **Variables** |  | Exp(B) (95% Confidence interval) | |
| --- | --- | --- | --- |
|  | **Items** | **Practice (Indoor)** | **Practice (Outdoor)** |
| **Sociodemographic characteristics** | **Gender (=Female)** | 0.957 (0.722, 1.269) | 1.109 (0.843, 1.459) |
|  | **Age** | 1.479 (1.171, 1.867) ** | 1.360 (1.083, 1.707) ** |
|  | **Marital status (=Unmarried or Single)** | 0.828 (0.579, 1.184) | 1.170 (0.812, 1.687) |
|  | **Location (=Urban area)** | 1.701 (1.050, 2.755) * | 1.084 (0.689, 1.705) |
|  | **Education** | 0.991 (0.768, 1.280) | 0.766 (0.596, 0.985) * |
|  | **Monthly income** | 0.867 (0.714, 1.054) * | 1.049 (0.862, 1.276) |
|  | **Occupation** | *Reference group - Unemployed* | |
|  | Student | 1.016 (0.545, 1.897) | 0.816 (0.450, 1.480) |
|  | Retired | 0.743 (0.449, 1.227) | 0.715 (0.441, 1.159) |
|  | Self-employed | 0.783 (0.395, 1.554) | 0.847 (0.424, 1.689) |
|  | Private sector | 0.814 (0.456, 1.451) | 0.697 (0.396, 1.226) |
|  | Public sector | 0.825 (0.449, 1.515) | 0.741 (0.410, 1.339) |
|  | Government sector | 0.473 (0.242, 0.923) | 0.741 (0.378, 1.452) |
| **Individual status** | **Infected (=No)** | 1.090 (0.751, 1.583) | 0.818 (0.573, 1.168) |
|  | **Family/friends Infected (=No)^a^** | 1.291 (0.715, 2.332) | 0.989 (0.546, 1.792) |
|  | **Trained or educated (=No)** | 0.722 (0.551, 0.947) * | 0.848 (0.649, 1.019) |
|  | **Risk Perception** | 1.499 (1.109, 2.027) ** | 1.323 (0.985, 1.776) |
|  | **Concerned about COVID-19** | 2.284 (1.814, 2.877) *** | 1.976 (1.575, 2.480) *** |
|  | **Knowledge regarding prevention** | 0.752 (0.529, 1.071) | 1.016 (0.718, 1.439) |
| **KAP** | **Knowledge** | 1.050 (0.992, 1.111) | 0.994 (0.941, 1.051) |
|  | **Attitude** | 1.338 (1.209, 1.482) *** | 1.451 (1.311, 1.605) *** |
| **Pearson Chi-Square** |  | 4501.956 | 4521.838 |
| **Log likelihood** |  | -1339.198 | -1403.494 |
| **CAIC** |  | 2873.456 | 3002.047 |
| **AIC** |  | 2728.396 | 2856.987 |
| **BIC** |  | 2848.456 | 2977.047 |

Note: AIC, Akaike information criterion; BIC, Bayesian information criterion; CAIC, Consistent Akaike information criterion.

a Eliminated “I don’t know” responses.

*p < 0.05, ** p < 0.01, *** p < 0.001.
